# Evaluation of the Ventral Hernia Working Group classification for long-term outcome using English Hospital Episode Statistics: a population study

**DOI:** 10.1007/s10029-021-02379-8

**Published:** 2021-03-13

**Authors:** J. D. Hodgkinson, G. Worley, J. Warusavitarne, G. B. Hanna, C. J. Vaizey, O. D. Faiz

**Affiliations:** 1 Department of Colorectal Surgery, St Mark’s Hospital and Academic Institute, Watford Road, Harrow, London, HA1 3UJ UK; 2 Department of Surgery and Cancer, Imperial College London, London, UK

**Keywords:** Ventral hernia, Incisional hernia, Recurrent hernia, VHWG classification

## Abstract

**Purpose:**

The Ventral Hernia Working Group (VHWG) classification of ventral/incisional hernia (IH) was developed by expert consensus in 2010. Subsequently, Kanters et al. have demonstrated the validity of a modified version of the system for predicting short-term outcomes. This study aims to evaluate the modified system for predicting hernia recurrence.

**Methods:**

Patients undergoing IH surgery (defined by OPCS codes) in the England Hospital Episode Statistics (HES) database, from 1997 to 2012, were identified. Baseline demographics at index hernia operation and episodes of further hernia surgery (FHS) were recorded. Risk factors for FHS were identified using cox regression and evaluated against the modified-VHWG grade using receiver-operating characteristics (ROC).

**Results:**

The final analysis included 214,082 index IH operations. Of these, 52.6% were female and mean age was 56.59 (SD15.9). An admission for FHS was found in 8.3% cases (17,714 patients).

Multi-variate cox regression revealed contaminated hernia (*p* < 0.0001), pre-existing IBD (*p* < 0.0001) and hernia comorbidity (*p* = 0.05) to be significantly related to long-term FHS. Classifying patients using these factors, according to the modified-VHWG classification, revealed that compared to Grade 1, the hazard ratio (HR) of FHS increased in Grade 2 (HR 1.19; *p* < 0.0001) and further increased in Grade 3 (HR 1.79; *p* < 0.0001). ROC analysis revealed the area under the curve to be 0.73 (95% CI 0.73–0.74).

**Conclusion:**

This analysis demonstrates the broad validity of the modified-VHWG classification in discriminating risk for FHS. Inclusion of pre-existing IBD as a factor defining Grade 2 patients would be recommended. This analysis is limited by the absence of certain factors within the HES database, such as BMI.

## Introduction

Hernia surgery encompasses a wide range of the general surgical workload. The incidence of incisional hernia following laparotomy is thought to be approximately 10% [1–5] and 10% of these hernia repairs will recur and will require further surgical repair [6]. Population level studies of incisional hernia surgery are rare. This is likely as a result of the large variation the scope and scale of hernia surgery.

Defining the complexity of incisional/ventral hernia surgery has proved difficult with multiple factors thought to play a role in complicating a hernia operation, such as patient comorbidity, the presence of contamination or the defect size. A number of attempts have been made to classify ventral hernia defect in order to define complexity and aid comparison between studies [7–9]. Although many of these were described a number of years ago, none has been taken up across the surgical community or successfully validated across all cases.

**Fig. 1:**
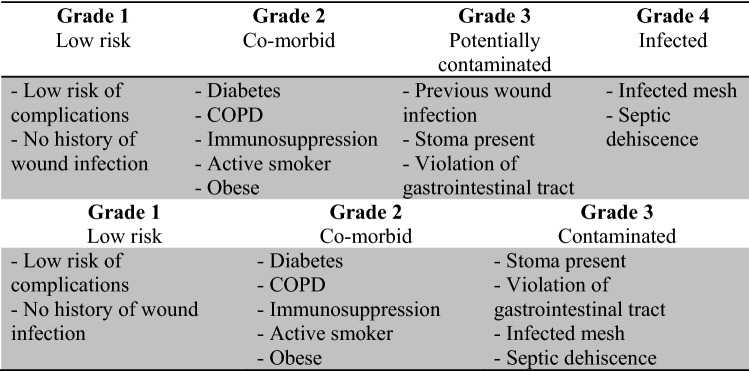
An overview of the Ventral Hernia Working Group (VHWG) Classification and the Kanters modified-VHWG classification

In 2010, the Ventral Hernia Working Group (VHWG) defined a simple grading system for ventral hernia surgery with the aim of grouping patients by increasing risk [10]. They graded defects from 1, clean defects in healthy patients, to 4, contaminated defects (Fig. 1). The overall validity of this system has never been proven.

A single-centre study by Kanters et al. aimed to validate the system for surgical site occurrence (SSO). They found the risk of a SSO was better predicted by combining the contaminated Grade 3 and 4 into a single group and stratifying these patients according to the centre for disease control (CDC) wound classification (Fig. 1) [11]. No study has demonstrated the validity of the VHWG grades for long-term outcomes, such as hernia recurrence, however, systematic review data have suggested that hernia recurrence is more likely in contaminated cases [12, 13].

This study aims to assess the scale of ventral/incisional hernia surgery in England using the hospital episode statistics (HES) database. These data will then be used to evaluate the ability of the VHWG grading system to predict long-term outcomes using comorbidity and operative data coded for in HES.

## Methods

### Defining the dataset

The HES database was set up in 1987 aiming to capture every hospital attendance, out-patient, emergency attendance and elective admission, across England for all patients. All operations carried out during the admission are coded using office of population censuses and surveys classification of interventions and procedures (OPCS code). Other diagnoses and comorbidities relevant to the admission are identified using the international classification of diseases (ICD)-10 classification system.

The HES database from 1st April 1997 to 31st March 2012 was evaluated. Ethical approval for use of the HES dataset was given by the London board (HRA NRES 2013: REC reference 13/LO/1235). All patients who underwent an operation for incisional and ventral hernia were identified using the OPCS codes in Table 1. All hospital admissions for these patients were then identified and isolated from the full HES database using the patients’ unique HES identification code. Cases were excluded if they were under the age of 18 at the time of their index hernia operation and if there were missing data within the index hernia admission episode.

**Table 1:** OPCS codes used to identify incisional/ventral hernia surgery in the HES database and ICD-10 and OPCS codes used to group patients according to the Ventral Hernia Working Group classification

Hernia procedure OPCS code	Comorbidity ICD-10 code	Concomitant GI tract surgery OPCS code
T25—primary repair of incisional hernia T26—repair of recurrent incisional hernia T27—primary repair of ventral hernia T28—abdominal wall reconstruction Y71.2—modifier for recurrent hernia	E10-14—diabetes mellitus J44—chronic obstructive pulmonary disease D80-84—immunodeficiency Y434—immunosuppressive medication K50-51—inflammatory bowel disease I25—chronic ischaemic heart disease I50—heart failure Q796—hypermobility/Ehlers–Danlos syndrome	G01–11, 26–29, 30–36, 40–41, 49, 50–53, 60–61, 63, 68–69, 70–79—small bowel operation H01–19, 29, 32–33—colon operation J19, 27.1–3, 29.1–2, 30.1–3, 34–36, 54.1, 56.1–3, 56.8–9—other GI tract operation

Hernia operations were retrospectively graded using the modified-VHWG classification system [11]. Grade 3 cases were identified using OPCS code for operations that involve violation of the GI tract at the same operation as the hernia repair (Table 1). These cases were then removed from the dataset resulting in the presence of only ‘clean’ cases. These were then divided into Grade 2 by identifying relevant hernia comorbidity defined by the grading system [diabetes mellitus (DM), chronic obstructive pulmonary disease (COPD), immunosuppression] using ICD-10 codes both at the time of hernia surgery and listed at a previous admission for the same case. The remaining cases, without comorbidity or concomitant GI tract violation, were classified as Grade 1.

Further relevant comorbidity coded for at the index hernia operation or admissions prior to the hernia repair, such as ischaemic heart disease (IHD), inflammatory bowel disease (IBD) and Ehlers–Danlos Syndrome, were also highlighted using ICD-10 codes (Table 1). Other demographics captured by HES, including index of multiple deprivation (IMD), emergency admission and planned day case admission, were identified.

Outcomes from the index hernia operation were identified from the HES database, including post-operative length of stay. Future hospital admissions were evaluated for further hernia operations, identified using the same OPCS codes. Time to further hernia surgery and number of further hernia operations were evaluated. The HES dataset was also combined with office of national statistics (ONS) data to define dates of death where relevant. In this way, 30- and 90-day post-operative mortalities were identified. Patients were censored either by the earliest applicable date; date of death (if relevant); date of first further hernia surgery; or the last HES date (31st March 2012).

### Statistical analysis

Statistical analysis was performed using SPSS (V20, IBM, New York, USA). Categorical demographic and outcome data are presented as numbers and percentages. Continuous data are expressed as a mean and standard deviation, if parametric, and a median, inter-quartile range and range, if non-parametric. Point prevalence was determined by calculating rates per 100,000 of the population based on the total population of England (ONS) for all hernia surgery and per 100 hernia operations for repairs in the presence of contamination.

Evaluation of the modified-VHWG grading system, as a stratification tool for hernia recurrence, was performed using cox regression analysis of time to censor. Variables were assessed for significance using uni-variate cox regression. Proportional hazards were assessed using log–log and Schoenfeld plots and those variables that did not meet the assumptions were re-evaluated with time-dependent co-variables. Factors found to be significant on uni-variate analysis (*p* < 0.01) were placed into a multi-variate cox regression model with backward-stepwise analysis based on likelihood ratio to determine to optimal factors for inclusion in the final grading system. Factors deemed significant for inclusion in the final model (inclusion *p* = 0.05; exclusion *p* = 0.10) were collated into a modified-grading system based on the original VHWG grade. Results are presented as unadjusted and adjusted hazard ratios. A survival curve, demonstrating time to further hernia surgery, for the final model for further hernia surgery was evaluated using receiver-operating characteristics.

## Results

### Baseline demographics at index hernia operation

The database search identified 221,537 index hernia operations with 2,816,939 hospital admissions over the time period. Cases with missing data were identified and excluded resulting in 214,082 cases included in the final analysis [7,455 excluded (3.37%)]. This is an average of 7.8 admissions per case, however, the distribution is skewed towards those with multiple comorbidities and not all admissions were related to the hernia surgery.

The majority of included patients were female (113,539, 52.6%) with a mean age of 56.59 (SD 15.9) and the majority of operations were performed in the 60–69 age category (21.9%). The hernia operation was planned as a day case procedure in 21.9% (46,798 patients) and performed as an emergency in 16.1% (34,456 patients) (Table 2).

**Table 2:** Baseline demographics of the whole cohort split by modified-VHWG grade

	Modified VHWG grade *N* (%)			Whole cohort *N* (%)
	1	2	3	Total
Sex (male)	81,271 (47.3)	9,905 (46.4)	10,257 (49.0)	101,433 (47.4)
Age (mean ± SD)	54.63 (15.8)	64.05 (12.2)	64.98 (14.7)	56.59 (15.9)
18–29	9,759 (5.7)	120 (0.6)	402 (1.9)	10,281 (4.8)
30–39	23,731 (13.8)	592 (2.8)	957 (4.6)	25,280 (11.8)
40–49	33,729 (19.6)	2,095 (9.8)	4,982 (9.5)	37,806 (17.7)
50–59	35,122 (20.4)	4,063 (19.0)	3,226 (15.4)	42,411 (19.8)
60–69	34,786 (20.2)	6,791 (31.8)	5,221 (24.9)	46,798 (21.9)
70–79	26,000 (15.1)	5,865 (27.5)	5,945 (28.4)	37,810 (17.7)
> 80	8,656 (5.0)	1,820 (8.5)	3,220 (15.4)	13,696 (6.4)
Hernia comorbidity	N/A	21,346 (100)	4,102 (19.6)	25,448 (11.9)
Diabetes	N/A	14,779 (69.2)	2,604 (12.4)	17,383 (8.1)
COPD	N/A	7,676 (36.0)	1,747 (8.3)	9,423 (4.4)
Immuno-deficiency	N/A	88 (0.4)	18 (0.0)	106 (0.0)
IHD	5,553 (3.2)	2,658 (12.5)	1,741 (8.3)	9,952 (4.6)
Pre-existing IBD	1,674 (1.0)	247 (1.2)	1,484 (7.1)	3,405 (1.6)
Crohn’s Disease	1,152 (0.7)	137 (0.6)	870 (4.2)	2,159 (1.0)
UC	522 (0.3)	110 (0.5)	614 (2.9)	1,246 (0.6)
Ehlers–Danlos	20 (0.0)	1 (0.0)	3 (0.0)	24 (0.0)
Contaminated hernia repair	N/A	N/A	20,953 (100)	20,953 (9.8)
Emergency operation	21,305 (12.4)	4,337 (20.3)	8,814 (42.1)	34,456 (16.1)
Planned as day case	53,695 (31.3)	3,053 (14.3)	463 (2.2)	57,211 (26.7)

*VHWG* Ventral Hernia Working Group, *IHD* ischaemic heart disease, *IBD* inflammatory bowel disease, *UC* ulcerative colitis

When the cohort was divided and classified according to the modified-VHWG grades, 80.2% (171,783) were Grade 1, 9.97% (21,436) were Grade 2 and 9.8% (20,953) were Grade 3. Hernia comorbidity was present overall in 11.9% (24,481 patients) (Table 2).

**Fig. 2:**
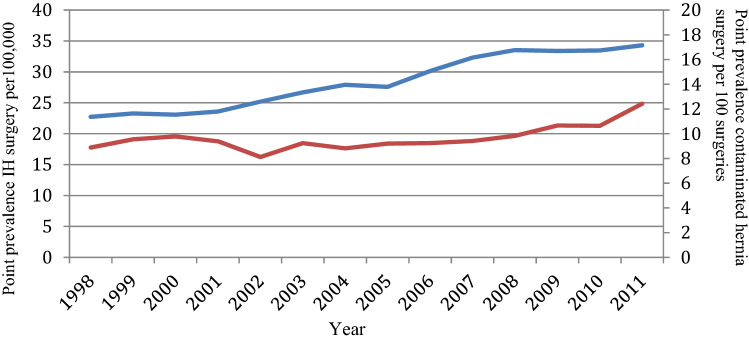
Point prevalence of incisional hernia (IH) surgery (blue) per 100,000 of the population and of contaminated hernia surgery per 100 hernia surgeries (red)

When evaluating the number of hernia operations performed in each complete year for the HES database (1998–2011), the point prevalence has increased from 22.7/100,000 in 1998 to 34.3/100,000 in 2011. Of these, the point prevalence of contaminated repairs has also increase over time, from 8.9/100 in 1998 to 12.4/100 in 2011 (Fig. 2).

### Hernia surgery outcomes

Median length-of-stay post-index hernia operation was 2 days (IQR 1–6; range 0–792). Thirty-day post-operative mortality was 1.4% (2,941 patients) and 90-day mortality was 2.1% (4,550 patients) for all cases. The short-term mortality rates increased across the modified-VHWG grades. Thirty-day mortality was 0.6% in Grade 1, 1.8% in Grade 2 and 7.3% in Grade 3. The same pattern was seen at 90-day mortality; 1.0% in Grade 1; 2.9% in Grade 2; and 10.6% in Grade 3 (Table 3).

**Table 3:** Outcomes from index hernia operation

	VHWG grade			Total (*N*, %)
	1 (*N*, %)	2 (*N*, %)	3 (*N*, %)	
LOS (days)	2	3	11	2
(Median; IQR; range)	(0–4; 0–564)	(1–7; 0–378)	(6–20; 0–792)	(1–6; 0–792)
30-day mortality	1,012 (0.6)	391 (1.8)	1,538 (7.3)	2,941 (1.4)
90-day mortality	1,703 (1.0)	625 (2.9)	2,222 (10.6)	4,550 (2.1)
Recurrent hernia surgery	13,889 (8.1)	1,566 (7.3)	2,259 (10.7)	17,714 (8.3)
Multiple hernia recurrences	2,412 (17.4)	260 (16.6)	468 (20.7)	3,140 (17.7)

Multiple hernia recurrences are presented as a proportion of the total hernia recurrences

*LOS* length of stay

An admission for further hernia surgery was found in 8.3% of the cases (17,714 patients). The highest numbers of further hernia operations were seen in Grade 3 cases (10.7%, 2,259 patients). The median number of further hernia operations was 1, with a range of 0–11. Of those who had a further hernia operation approximately 80% did not need a second further hernia surgery. In Grade 1, 17.4% of patients needed multiple further operations, 16.6% in Grade 2 and 20.7% in Grade 3 (Table 3).

### Evaluation of the modified-VHWG classification for stratification of patients by likelihood of further hernia surgery

The modified-VHWG grading system was evaluated as a stratification tool using cox regression. Factors identified within HES thought to be associated with an increased risk of recurrence were evaluated with uni-variate analysis. Only the presence of hypermobility pre-operatively was found to be not significant (HR 2.53; 95% CI 0.95–6.74; *p* = 0.06). Proportion hazards assumptions were violated by sex, hernia comorbidity, IHD pre-operatively, pre-existing inflammatory bowel disease (IBD), contaminated hernia repair and emergency hernia repair. No violation was found with patient age.

Uni-variate cox regression was then repeated with time-dependent co-variables for the relevant factors. Patient sex and emergency hernia surgery were then found to not be significantly associated with further hernia surgery (*p* = 0.14 and *p* = 0.29, respectively). The remaining factors were entered into the multi-variate model to determine the variables with the most influence on the likelihood of further hernia surgery (Table 4).

**Table 4:** Unadjusted and adjusted proportionate hazard cox regression model evaluating factors linked to further hernia surgery

	Unadjusted analysis		Adjusted multi-variate analysis	
	Hazard ratio (95% CI)	*p* value	Hazard ratio (95% CI)	*p* value
Age	1.002 (1.001–1.003)	< 0.0001	0.997 (0.994–1.001)	0.45
Sex (male)	1.03 (0.99–1.08)	0.14		
Hernia comorbidity	1.09 (1.02–1.17)	0.01	1.05 (1.00–1.10)	0.05
IHD	1.17 (1.06–1.30)	0.003	1.05 (0.98–1.10)	0.18
IBD	1.76 (1.55–2.01)	< 0.0001	1.52 (1.38–1.67)	< 0.0001
Hypermobility	2.53 (0.95–6.74)	0.06		
Contamination	1.78 (1.67–1.89)	< 0.0001	1.69 (1.62–1.77)	< 0.0001
Emergency surgery	1.03 (0.97–1.09)	0.29		

*IHD* ischaemic heart disease, *IBD* inflammatory bowel disease

The multi-variate model retained contaminated hernia repair, pre-existing IBD and hernia comorbidity as significant factors for the risk of further hernia surgery (Table 4). Given pre-existing IBD was found to be a significant factor in the likelihood of further hernia surgery, it was included in the modified-VHWG classification as a comorbidity that defines a Grade 2 patient. Survival analysis including this additional modification to the VHWG grading system revealed a significant difference between the three grades in the prediction of further hernia surgery (Table 5). Compared to Grade 1, the hazard ratio (HR) of further hernia surgery increased in Grade 2 (HR 1.19; 95% CI 1.13–1.25; *p* < 0.0001) and further increased in Grade 3 (HR 1.79; 95% CI 1.72–1.88; *p* < 0.0001).

**Table 5:** Cox regression analysis of the St Mark’s modified Ventral Hernia Working Group (VHWG) grading system for its ability to discriminate between patients risk of further hernia surgery

St Mark’s modified VHWG grade	Patient number (*N*, %)	Further hernia surgery (*N*, %)	Patient years of follow-up	Recurrences/patient year of follow-up	Hazard ratio (95% CI)	*p* value
1	170,109 (79.5)	13,661 (8.0)	918,713	0.015	1	
2	23,020 (10.8)	1,794 (7.8)	83,232	0.022	1.19 (1.13–1.25)	< 0.0001
3	20,953 (9.8)	2259 (10.8)	72,799	0.031	1.79 (1.72–1.88)	< 0.0001

**Fig. 3:**
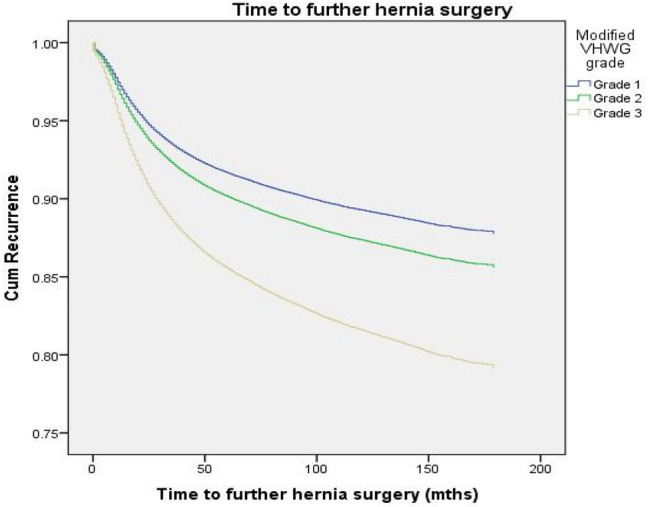
Survival curve demonstrating the ability of the St Mark’s modified Ventral Hernia Working Group (VHWG) grading system to discriminate between the risk of further hernia surgery

**Fig. 4:**
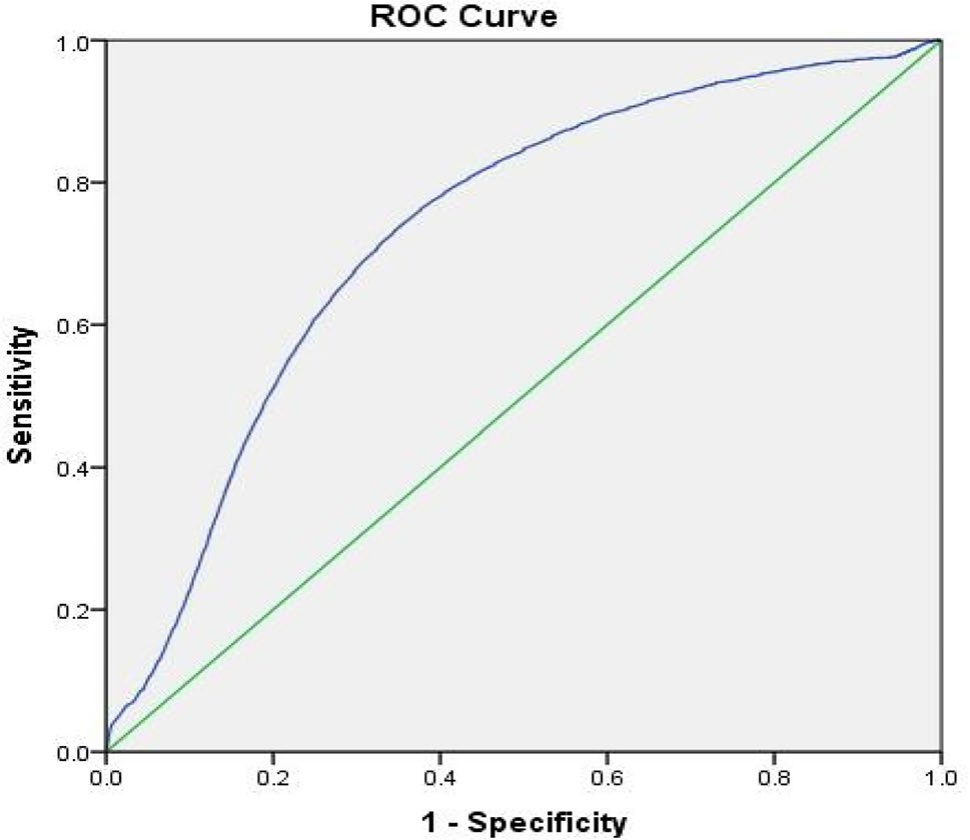
Receiver-operating characteristic curve assessing the validity of the St Mark’s modified Ventral Hernia Working Group (VHWG) grading system (AUC 0.73; 95% CI 0.728–0.736)

Survival curves for each Grade demonstrated a likelihood of further hernia surgery of 11% in Grade 1, 14% in Grade 2 and 20% in Grade 3 at 15-year follow-up (Fig. 3). Interestingly, the majority of further hernia surgery took place in the first 3 years from index operation (6% in Grade 1, 8% in Grade 2 and 12% in Grade 3). Analysis with ROC revealed the area under the curve to be 0.73 (95% CI 0.73–0.74) demonstrating a moderate ability to discriminate between patients (Fig. 4).

## Discussion

This study demonstrates the point prevalence of ventral/incisional hernia surgery has increased over time, as has the point prevalence of contaminated cases. The incidence of further hernia surgery is approximately 10%, which is consistent with previously published rates. Of those patients who had further hernia surgery, 20% went on to have multiple further hernia operations. This is double the rate seen in previously published data and suggests hernia recurrence following hernia repair is a bigger problem than previously realised. It is the first study to demonstrate the modified-VHWG classification system is a valid tool to stratify patients by risk of their likelihood of requiring further hernia surgery and clearly shows contamination at the time of hernia surgery as the largest discriminating factor.

When evaluating the risk of further hernia surgery some risk factors implicated in further hernia surgery are consistent with those previously described in the literature including grouped hernia comorbidity (diabetes, COPD and immunosuppression) and contaminated hernia surgery. Inflammatory bowel disease is shown here as a novel risk factor for further hernia surgery (*p* < 0.0001). This could be consistent with the likelihood that patients with inflammatory bowel disease are more likely to undergo more abdominal procedures [14–16], however, further analysis of pre-existing IBD as a factor in the recurrence of incisional hernia would be interesting.

While the modified-VHWG classification has been shown to adequately risk stratify patients on the basis of wound outcome [11], no study to date has demonstrated its use in the stratification of long-term hernia recurrence outcomes. These data suggest the addition of inflammatory bowel disease as a relevant hernia comorbidity related to recurrence would improve the accuracy of the model as a tool for stratifying patients on the based of likelihood for long-term further hernia surgery with moderate accuracy based on ROC analysis.

It is noted that the absolute proportion of Grade 2 patients who had further hernia surgery was lower than in Grade 1 (7.8% vs 8.0%, respectively). This is the result of a shorter time to recurrence in this group as demonstrated by the higher number of recurrences per patient year of follow-up.

The principle limitation of this analysis is the use of further hernia surgery as an outcome measure for recurrence. As discussed by Muysoms et al. [9], the hierarchy of quality of follow-up in hernia surgery places further surgery as the lowest quality of evidence based on the likelihood of underestimating recurrence rates. This would be consistent in the presented data as reported rates of incisional hernia recurrence are estimated to be approximately 10% (range 5–45%) [17–19] where as our observed rate of further surgery is only 8.3%. While this is likely an under estimate of the overall rate of recurrence, collecting prospective data, such as this, on this scale with accurate diagnosis of recurrent hernia, based on imaging finding (as per Muysoms et al.), is unrealistic and this analysis is likely to be accurate in its validation, as it is possible to be, given the cohort size.

It is also interesting to note that length of stay, 30- and 90-day mortalities were higher in modified Grade 3 cases. This is likely related to the concomitant operations that were carried out at the time of hernia increasing procedure complexity, and therefore, mortality. This could also be due to the factor a much higher number of the Grade 3 cases were carried out in the emergency setting. However, it must be noted that this group carries a significant risk of post-operative mortality when counselling patients and planning for surgical intervention.

The other limitations of this study are consistent with any large retrospective administrative database analysis. There is a possibility of coding error, particularly in relation to the accuracy of hernia operation OPCS codes. The index hernia operation was taken to be the first operation within the dataset for an incisional or ventral hernia. It was shown that although some of these were coded as primary ventral hernias the patient had undergone (sometimes multiple) previous abdominal operations. Given the incidence of incisional hernia is 10% and the rate of recurrent hernia is around 20% following repair the chance of there being a large enough number of patients to effect the analysis in a cohort of this size is low. As well as this there is no way of distinguishing between midline hernias and lateral abdominal wall hernias in the OPCS codes and different incisions have been shown to have different rates of incisional and recurrence hernia [6]. Again the number of patients affected and the differences in overall incisional and hernia recurrence rates are likely to be small, and therefore, unlikely to affect the final results.

Finally, there are a number of co-factors implicated in the development of incisional hernia and the rate of hernia recurrence that are not coded for within HES. While size of defect is thought to be important the largest two missing factors are smoking status and body mass index (BMI). These two factors would increase the number of patients classified as Grade 2 cases and probably explains the limited size of this cohort as a proportion of the overall case mix by comparison to previous studies [11, 20]. Given the difference in long-term further hernia surgery rates between Grade 1 and 2 is significantly different and it is likely the inclusion of smoking and BMI would only widen this gap, it is relatively safe to assume the validation of the modified-VHWG classification is accurate. The question of whether the inclusion of these factors could aid in further sub-classifying the contaminated Grade 3 patients remains to be answered.

This study has demonstrated the point prevalence of incisional/ventral hernia surgery is increasing over time as well as the proportion of those that are contaminated. It has shown the broad validity of the modified-VHWG grading system in stratifying patients by risk of further hernia surgery. In addition, we have demonstrated pre-existing IBD to be a significant risk factor for long-term further hernia surgery and would recommend it should be included in the definition of Grade 2 patients.

## Abbreviations

VHWG: Ventral Hernia Working Group
SSO: Surgical site occurrence
CDC: Centre for disease control
HES: Hospital Episode Statistics
OPCS code: Office of population censuses and surveys classification of interventions and procedures
ICD: International classification of diseases-10
DM: Diabetes mellitus
COPD: Chronic obstructive pulmonary disease
IHD: Ischaemic heart disease
IBD: Inflammatory bowel disease
IMD: Index of multiple deprivation
ONS: Office of national statistics
BMI: Body mass index
ROC: Receiver-operating characteristics
HR: Hazard ratio
FHS: Further hernia surgery
